# Identification of the *NUP98-PHF23* fusion gene in pediatric cytogenetically normal acute myeloid leukemia by whole-transcriptome sequencing

**DOI:** 10.1186/s13045-015-0167-8

**Published:** 2015-06-12

**Authors:** Marco Togni, Riccardo Masetti, Martina Pigazzi, Annalisa Astolfi, Daniele Zama, Valentina Indio, Salvatore Serravalle, Elena Manara, Valeria Bisio, Carmelo Rizzari, Giuseppe Basso, Andrea Pession, Franco Locatelli

**Affiliations:** Department of Pediatrics, “Lalla Seràgnoli” Hematology-Oncology Unit, University of Bologna, Bologna, Italy; Department of Paediatric Haematology, University of Padova, Padova, Italy; Giorgio Prodi Cancer Research Centre, University of Bologna, Bologna, Italy; Department of Pediatrics, San Gerardo Hospital, University of Milano-Bicocca, Monza, Italy; Department of Pediatric Hematology-Oncology, IRCCS Ospedale Bambino Gesù, Roma - University of Pavia, Pavia, Italy

**Keywords:** *NUP98* gene fusions, Pediatric acute myeloid leukemia, PHD domain

## Abstract

The genomic landscape of children with acute myeloid leukemia (AML) who do not carry any cytogenetic abnormality (CN-AML) is particularly heterogeneous and challenging, being characterized by different clinical outcomes. To provide new genetic insights into this AML subset, we analyzed through RNA-seq 13 pediatric CN-AML cases, corroborating our findings in an independent cohort of 168 AML patients enrolled in the AIEOP AML 2002/01 study. We identified a chimeric transcript involving *NUP98* and *PHF23*, resulting from a cryptic t(11;17)(p15;p13) translocation, demonstrating, for the first time, that *NUP98-PHF23* is a novel recurrent (2.6 %) abnormality in pediatric CN-AML.

## Findings

Childhood acute myeloid leukemia (AML) is a heterogeneous disease with current survival rates of approximately 60–70 %. Cytogenetics, recurrent molecular abnormalities, and early response to treatment are the main factors influencing outcome [1]. However, around 20 % of pediatric AML do not carry any known cytogenetic abnormality (cytogenetically normal-AML or CN-AML). In order to shed light on this subgroup we performed whole-transcriptome sequencing (WTS) in 13 pediatric CN-AML cases, corroborating relevant findings in an independent cohort of 168 cases.

**Fig. 1:**
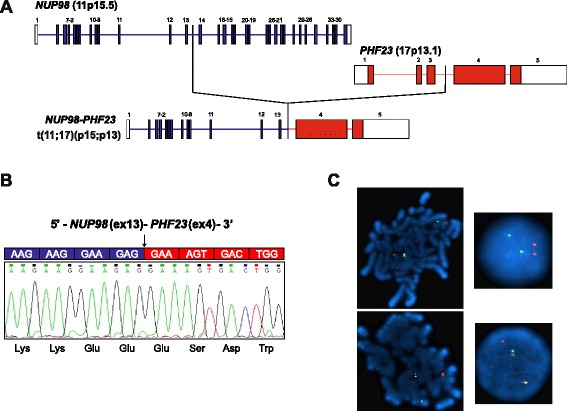
Identification of *NUP98-PHF23* in pediatric CN-AML. **a** Schematic representation of *NUP98-PHF23* fusion identified by RNA-seq in pediatric CN-AML. Fusion occurs between exon 13 of *NUP98* and exon 4 of *PHF23*. **b** Electropherogram from Sanger sequencing of the region surrounding the breakpoint confirmed the in-frame fusion. A *black arrow* indicates the fusion breakpoint, predicted sequence of the fusion protein is shown. **c** FISH analysis was performed on metaphases and interphase cells using three BlueFISH probes (BlueGnome Ltd., Cambridge), according to the manufacturer’s instructions. BAC clones RP11-120E20 and RP11-348A20 (red) were used to probe the *NUP98* gene on chromosome 11, while the BAC clone RP11-542C16 (*green*) was used to target the *PHF23* gene on chromosome 17. Normal metaphases (*upper left*) and interphase nuclei (*upper right*) showed two *red signals* representing normal copies of *NUP98* and two *green signals* representing normal copies of *PHF23*. Abnormal metaphases (*lower left*) and interphase cells (*lower right*) containing the *NUP98-PHF23* fusion gene showed one *red* (*NUP98*), one *green* (*PHF23*) and one *yellow* fusion signal, which represents the juxtaposition of the translocated portions of the two genes

Sequencing was performed on a HiScanSQ sequencer (Illumina), and bioinformatic analysis was performed as previously described [2]. In 2 (CN-AML_54, CN-AML_66) out of 13 cases analyzed, we identified a chimeric transcript involving nucleoporin 98 kDa (*NUP98*) and PHD finger protein 23 (*PHF23*) genes, resulting from a cryptic translocation t(11;17)(p15;p13) (Fig. 1a and Table 1). In both cases, we identified an *in-frame* fusion between *NUP98* exon 13 and *PHF23* exon 4 (Fig. 1b). To date, the cryptic translocation t(11;17)(p15;p13) has been described only once in an adult AML patient [3], but never in a pediatric AML cohort. Different from what was previously reported by Reader and colleagues [3], in this study the recurrent breakpoint in *PHF23* was always identified at the beginning of exon 4 and not within it (Fig. 1a and b).

**Table 1:** Clinical features of pediatric CN-AML patients harboring the *NUP98-PHF23* fusion gene

Id	Age, years	Gender	WBC, x 10^9^/L	FAB	BM blast, % at diagnosis	Extramedullary involvement	HSCT (type)	CR after induction therapy	Relapse (site)	Disease-free duration (months)	Survival duration (months)
CN-AML_54^a^	2.9	M	187	M1	90	No	Yes (AUTO)	Yes	Yes (BM)	5	30^b^
CN-AML_66^a^	9.0	M	1.2	M0	70	No	Yes (MUD)	Yes	–	65	66
CN-AML_3	9.7	M	6.9	M4	40	No	Yes (MUD)	Yes	–	40	41
CN-AML_4	7.0	M	1.8	M5A	54	No	Yes (AUTO)	Yes	–	103	104

*AUTO* autologous, *CR* complete remission, *HSCT* hematopoietic stem cell transplantation, *MUD* matched unrelated donor, *BM* bone marrow, *WBC* white blood cells

^a^patients identified by RNA-seq

^b^dead patient

To assess the incidence of *NUP98-PHF23* fusion in pediatric CN-AML, we examined through RT-PCR analysis and Sanger sequencing a validation cohort of 168 AML children enrolled in the AIEOP AML 2002/01 study [4]; one-hundred thirty-nine patients (76 males and 63 females, median age at diagnosis 7.7 years, range 17 days to 17.9 years) were negative for known recurrent genetic abnormalities involving *MLL*, *CBFB*, and *FLT3*, while the remaining 29 patients (15 males and 14 females, median age at diagnosis 11.8 years, range 3 to 17.4 years) harbored internal tandem duplication of *FLT3* (*FLT3-ITD*), this latter abnormality being chosen because we previously reported a strong association between *NUP98-NSD1* rearrangement and *FLT3-ITD* [5]. With the exception of FAB M3 (acute promyelocytic leukemia), all the FAB types were represented in the validation cohort. RNA was extracted from fresh bone marrow at diagnosis, and multiplex RT-PCR was used. Sequencing by Sanger method was applied to all cases positive by PCR to *NUP98-PHF23* fusion gene. Overall, 2 out of 139 CN-AML cases were found to harbor *NUP98-PHF23* (Table 1). *NUP98-PHF23* was not found in any patient harboring *FLT3-ITD*. Fluorescence in-situ hybridization confirmed the cryptic chromosomal translocation t(7;11)(p15;p13) leading to the fusion between *NUP98* and *PHF23* in all cases (Fig. 1c).

So far, many NUP98-rearrangements have been found to be associated with both de novo and therapy-related AML but also with T-cell acute lymphoblastic leukemia with over 28 different partner genes [6]. Recently, the fusion *NUP98-JARID1A* has been described to be a recurrent event in pediatric acute megakaryoblastic leukemia (11 %), with a distinct *HOX* gene-expression pattern [7].

Conversely, chromosomal rearrangements and/or mutations of *PHF23* have never been previously described in children with AML. Located on the reverse strand of 17p13.1, *PHF23* encodes for a protein containing a plant homeodomain (PHD) finger [8] involved in chromatin remodeling [3]. Expression of *NUP98-PHF23* has been demonstrated to impair the differentiation of myeloid progenitor cells and promote leukemia development in vitro and in vivo [8–10]. Cells expressing *NUP98-PHF23* are sensitive to disulfiram, an FDA-approved drug, demonstrating the feasibility of targeting this oncoprotein [9].

In summary, we identified, for the first time in childhood AML, a *NUP98-PHF23* fusion, demonstrating that this genomic aberrancy is not exceptional (tentative frequency of 2.6 %) in pediatric CN-AML. These findings enforce the role of epigenetic regulators in pediatric AML and suggest novel therapeutic targets for this disease.
